# Magnetic resonance imaging in the diagnosis of trigeminal neuralgia: a systematic review of the imaging protocol and diagnostic accuracy

**DOI:** 10.1007/s00330-025-12141-8

**Published:** 2025-11-27

**Authors:** Dylan Henssen, Max van Grinsven, Kris Vissers, Johan van Goethem

**Affiliations:** 1 Department of Medical Imaging, Radboudumc, Nijmegen, The Netherlands; 2 Department of Nuclear Medicine, Leipzig University Hospital, Leipzig, Germany; 3 Department of Anesthesiology, Palliative Medicine and Pain Medicine, Radboudumc, Nijmegen, The Netherlands; 4 Department of Radiology, Sengkang General Hospital, Singapore, Singapore

**Keywords:** Magnetic resonance imaging, Trigeminal neuralgia, Classical trigeminal neuralgia, Systematic literature review

## Abstract

**Objectives:**

Trigeminal neuralgia (TN) is a severe pain disorder for which MRI helps to identify the anatomical causes of pain. However, imaging protocols vary widely, and a plethora of imaging features that might contribute to different forms of TN have been described. This review aims to (1) summarize current evidence on MRI techniques and their diagnostic performance, and (2) evaluate imaging markers that improve our understanding of TN pathophysiology and support clinical decision-making regarding surgical or interventional treatment.

**Materials and methods:**

A comprehensive literature search was performed in PubMed, EMBASE, and the Cochrane Library databases, yielding 1441 studies. After applying strict inclusion criteria, 140 articles were included, and three papers were added from cross-referencing.

**Results:**

The review revealed that advanced MRI sequences (e.g., 3D CISS, 3D FLASH, 3D FIESTA) improve the visualization of neurovascular conflicts. Imaging at 3.0 T was found to be superior to 1.5 T for detecting small vessels responsible for nerve compression. However, distinguishing clinically significant neurovascular conflicts from asymptomatic neurovascular contacts remains a challenge. In secondary TN, MRI reliably detects lesions related to multiple sclerosis and other pathological conditions. Diffusion tensor imaging (DTI) has shown potential in identifying microstructural changes in the trigeminal nerve, further supporting its diagnostic use.

**Conclusions:**

Although MRI remains an essential tool in diagnosing TN, limitations in its diagnostic accuracy highlight the need for more homogeneous study designs and further refinement of imaging protocols and interpretation criteria.

**Key Points:**

***Question*** *Accurate identification of the anatomical cause of trigeminal neuralgia is clinically important, but variable MRI protocols result in inconsistent diagnostic accuracy.*

***Findings*** *Advanced MRI sequences and higher field strength improve visualization of neurovascular conflict, although interobserver variability and limited clinical correlation remain major challenges.*

***Clinical relevance*** *Standardized magnetic resonance imaging protocols and clearer interpretation criteria may enhance diagnostic confidence and assist treatment planning in patients with suspected trigeminal neuralgia, particularly when surgical intervention is considered to relieve severe, medication-resistant facial pain.*

## Introduction

Trigeminal neuralgia (TN) comprises a group of debilitating facial pain disorders, characterized by short-lasting, unilateral, electric shock-like episodes triggered by innocuous stimuli. According to the International Classification of Headache Disorders third edition (ICHD-3) criteria, TN can be either classical (~75%), secondary (~15%) or idiopathic TN (~10%). In classical TN, pain arises from neurovascular compression of the trigeminal root, especially at the root entry zone. Secondary TN results from identifiable pathologies, such as multiple sclerosis. Idiopathic TN is diagnosed when no structural cause or neurovascular compression can be found [1, 2]. Although the substrate of these pains can be detected in some patients, it must be stressed that the underlying cause is not always clear [3].

MRI has become increasingly important in evaluating TN, particularly classical TN, as high-resolution imaging can visualize neurovascular contact and nerve distortion [2]. Advanced techniques such as 3D T2-weighted sequences, diffusion tensor imaging (DTI), and volumetric analyses have improved our ability to detect microstructural abnormalities in the trigeminal nerve. These modalities offer potential in differentiating between TN subtypes, identifying pain generators, and guiding treatment decisions.

Despite these advances, there is no consensus on which MRI parameters are most clinically relevant in TN. Variability in imaging protocols, lack of standardization, and heterogeneous study designs have made it difficult to compare findings across studies. Furthermore, it remains unclear whether imaging should play a routine role in diagnosis or primarily support clinical findings in complex cases [4–7].

This review aims to systematically assess the existing literature on MRI-based diagnostics in TN, with a particular focus on classical TN. We examine imaging protocols, morphometric and microstructural findings, reader variability, and the diagnostic workup of secondary TN, including TN associated with multiple sclerosis. Through this review, we aim to provide clarity on the diagnostic utility of MRI in TN and highlight areas requiring further standardization and research.

## Materials and methods

The study was registered with the Prospective Register of Systematic Reviews (PROSPERO, CRD42020209938). Screening of literature for this systematic review followed the Preferred Reporting Items for Systematic Reviews and Meta-Analyses (PRISMA) guidelines, using Ovid EMBASE, Ovid MEDLINE, and Cochrane Library as databases. The literature search strategy involved the use of keywords including “molecular imaging,” “magnetic resonance imaging,” “multiparametric magnetic resonance imaging,” “trigeminal neuralgia,” “facial pain,” “trigeminal neuropathic pain” and related terms. Searches were performed until May 2025.

All retrieved articles were added to Rayyan.ai (https://www.rayyan.ai/) for further screening. After removing duplicates, a radiologist-nuclear medicine physician, holding a PhD in neuro-imaging (D.H.), and a graduate student (M.v.G.) screened all retrieved studies. Any conflicting assessments were resolved by discussion. The first step of the literature assessment comprised screening on title and abstract to exclude papers that were deemed non-relevant as they did not focus on the role of imaging in TN. After this first round of reviewing, a second round followed. This second round of reviewing comprised a full-text review of the remaining articles. This was also done by the two researchers (D.H. and M.v.G.) independently. Again, conflicts were resolved by discussion. Full-text analysis was based on predefined exclusion criteria. The criteria for exclusion comprised: (1) Abstract only, (2) Not an original article, (3) Not published in English, (4) No discussion on the role of medical imaging in TN, (5) No investigation of diagnoses that are classified as TN following the ICHD-3 criteria, (6) No human subjects and (7) No case reports (papers with less than five patients suffering from TN were excluded as well). Although review articles could not be included in this systematic review, all retrieved literature reviews were assessed, and the literature list was examined for cross-referencing purposes.

Data extraction was performed by a radiology resident holding a PhD in neuro-imaging (D.H.). Studies were reviewed twice, and disagreements were resolved by regular discussions until consensus was reached. Extracted data included: (1) Article characteristics (title, authors, publication data), (2) Specifications of TN diagnosis following the ICHD-3 criteria (diagnosis, number of patients), (3) Specifications regarding the medical imaging protocol (e.g., MRI sequences, use of contrast agent).

Furthermore, the risk of bias of the included diagnostic studies was assessed using the Quality Assessment of Diagnostic Accuracy Studies 2 (QUADAS-2) tool was used to assess the risk of bias [8].

## Results

**Fig. 1:**
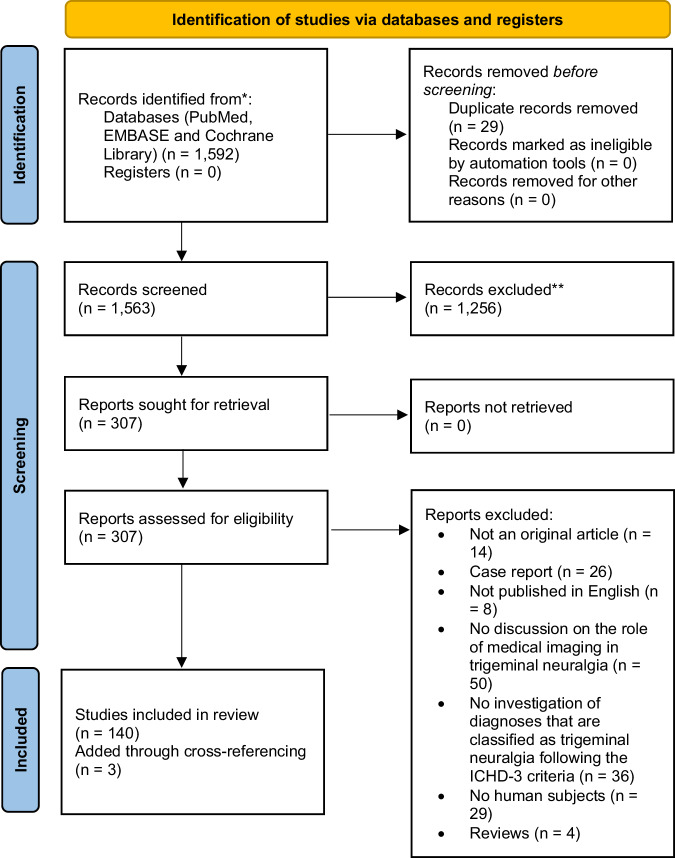
PRISMA 2020 flow diagram. * Consider, if feasible to do so, reporting the number of records identified from each database or register searched (rather than the total number across all databases/registers). ** If automation tools were used, indicate how many records were excluded by a human and how many were excluded by automation tools. From: Page et al [153]

In total, 1592 articles were retrieved. After removing 29 duplicates, the remaining 1563 articles were screened on title and abstract. This resulted in the exclusion of 1256 articles. Using the predefined exclusion criteria, the remaining 307 articles were assessed. After full-text review, 167 articles could be excluded, resulting in 140 studies that met the eligibility criteria of this review. Assessment of the bibliography of the retrieved literature reviews resulted in the inclusion of three additional papers. The flowchart of the literature screening process is provided in Fig. 1.

### Classical TN

#### How do imaging protocols and magnetic field strength influence the diagnostic accuracy of finding a neurovascular conflict?

A variety of studies investigated the role of different MRI sequences in identifying a neurovascular conflict [9–33]. See Table 1 for a detailed overview of the included studies and their specific recommendations. High-resolution 3D T2-weighted MRI sequences such as CISS, FIESTA, and DRIVE are most effective for detecting a neurovascular conflict in classical TN [14, 30, 34], especially when combined [18, 31]. The range of reported scan parameters in these studies are provided here: TR: 4.4–12.3 ms, TE: 1.4–5.9 ms, Flip angle: 40–70°, Slice thickness: 0.3–0.9 mm (isotropic), Field of view: 160 × 160–200 × 200 mm, Matrix: 224 × 224 or higher [14, 18, 30, 31, 34]. Use of 3-T scanners generally improves visualization [35–37], though protocol optimization is more critical than field strength alone. One study suggested that the use of a susceptibility-weighted imaging sequence could improve the visualization of offending veins [38]. The discussed papers showed overall a low risk of bias and low applicability concern (Table 2) (Recommendation 1 + 4; Table 3).

**Table 1:** Overview of included studies investigating different MRI protocols for the diagnosis of classical trigeminal neuralgia

Author (year)	Trigeminal neuralgia population	MR sequences	Proportion with NVC identified with imaging	Major findings
Yamakami et al [30]	*N* = 14 (3 males, 42–73 years)	1.5T, 3D CISS, 3D FISP	6/14 (3D FISP) 14/14 (3D CISS)	3D CISS was significantly better than 3D FISP for delineating anatomic detail and abnormal neurovascular relationships that could cause trigeminal neuralgia.
Akimoto et al [9]	*N* = 29 (10 males, 22–78 years)	1.5T, 3D CISS, 3D FISP	23/24	The combination of two types of 3D magnetic resonance images (3D CISS and 3D FISP) is very useful for creating preoperative simulations and in deciding whether to perform surgery in patients with trigeminal neuralgia.
Patel et al [24]	*N* = 92	1.5T, 3D MRA, 0.1 mmol/kg Gadopentetic acid	76/92	Based on the surgical findings, the sensitivity of MRA was 90.5% and the specificity was 100%.
Zerris et al [33]	*N* = 25	1.5T, CISS + FLASH*	24/25	The CISS/3D-Flash fusion imaging has become the preferred imaging method at the authors’ institutions during GKS for trigeminal neuralgia. It affords the best visualization of the trigeminal nerve, surrounding vasculature, and the precise location of vascular compression.
Chávez et al (2005)	*N* = 15 (5 males, 24–83 years)	1.5T, 3D FIESTA	N/A	The 3-D FIESTA sequence successfully demonstrated the trigeminal complex (root entry zone, trigeminal ganglion, rootlets, and vasculature) in 14 patients (93.33%).
Anderson et al [10]	*N* = 48 (15 males, mean age of 55 years)	1.5T, 3D TOF MRA, 3D T2 FSE, 3D SPGR 0.2 mmol/kg Gadolinium pentetate	42/48	Of the six cases in which no contact with the symptomatic nerve was visualized radiologically, four showed positive contact at microdissection (false negatives). There were no false-positive results. Overall, the sensitivity of 3D TOF MR angiography and 3D Gad imaging in visualization of neurovascular contact on the symptomatic nerve was 91% (42/46). The specificity was 100% (2/2).
Miller et al [20]	*N* = 18 (7 males, 26–80 years)	3T, 3D bFFE, 3D TOF MRA, 3D SPGR 0.1 mmol/kg Gadolinium DTPA	15/16	Combining BFFE with MR angiography and Gd-enhanced MR images capitalizes on the advantages of both techniques, enabling MR angiography and contrast-enhanced MR imaging discrimination of vascular structures at BFFE resolution.
Miller et al [21]	*N* = 30 (13 males, mean age of 54.8 years)	3.0T, 3D bFFE, 3D TOF MRA	17/30 arterial NVC 10/30 venous NVC	Trigeminal NVC occurs in asymptomatic patients but is more severe and more proximal in patients with trigeminal neuralgia.
Ni et al [23]	*N* = 33 (14 males, 35–76 years)	3.0T, 3D FSPGR Gadopentetic acid*, 3D TOF MRA	29/33	Enhanced 3D FSPGR in combination with 3D TOF MRA revealed contact or compression of vessels with the symptomatic nerve in 29 of 33 patients (88%).
Satoh et al [27]	*N* = 66 (22 males, 34–87 years)	1.0T or 1.5T, 3D T2 FSE, 3D TOF MRA	37/40 (40 cases underwent MVD)	The intraoperative findings disclosed a close similarity to the simulation in the anatomic relationship of the NVC. In the remaining 3 patients, the NVC was not observed in either the preoperative simulation images or the intraoperative findings. Consequently, in the 40 surgical patients, the preoperative MR images showed 100% correlation with the surgical findings regarding the presence or absence of the NVC.
Leal et al [76]	*N* = 40 (15 males, 22–79 years)	3.0T, T2-DRIVE, 3D TOF MRA, 3D T1w+C*	37/40	For the prediction of NVC, image analysis corresponded with surgical findings in 39 cases. Of the 3 patients in whom image analysis did not show NVC, 2 did not have NVC at the time of intraoperative observation.
Cha et al [11]	*N* = 66 (29 males, 27–79 years)	3.0T, T2 VISTA and FLAIR VISTA, 3D TOF MRA	63/66	MPR of T2 VISTA and FLAIR VISTA fusion imaging is useful in the detection of NVC in patients with vascular contact on the ipsilateral side was 63/66.
Vergani et al [28]	*N* = 67 (31 males, 32–84 years)	1.5T, 3D TOF MRA, T2 DRIVE	61/92	Preoperative MRI sensitivity was 96%, while specificity was 75% (1 false positive among 4 patients with negative intraoperative findings). The predictive value of a positive MRI/MRA was 98%, while the predictive value of a negative MRI/MRA was 50%
Zeng et al [32]	*N* = 37 (21 males, 26–81 years)	3.0T, 3D FIESTA, 3D TOF MRA	35/37	3D FIESTA in combination with MRA identified surgically verified neurovascular contact in 35 of 36 symptomatic nerves. The offending vessel (artery or vein) was correctly identified in 94.4% of patients, and agreement between preoperative MRI visualization and surgical findings was excellent (k = 0.92; 95% confidence interval, 0.67–1.00).
Lee et al [17]	*N* = 257 (97 males, mean age of 55.7 years)	3.0T, 3D bFFE, 3D TOF MRA	156/257	The sensitivity of imaging as a predictor of NVC for both 1 and 2 was 96%. The specificity of imaging findings for patients with 1 and 2 was 90% and 66%, respectively.
Maarbjerg et al [19]	*N* = 135 (53 males, mean age of 60.1 years)	3.0T, T2w GRASE, 3D TOF MRA, 3D bFFE	71/135	NVC is much more common in men than in women, indicating that women more often have other etiological factors contributing to it. Second, we find that severe NVC is not associated with age, duration of disease, concomitant persistent pain or sensory abnormalities at bedside examination.
Docampo et al [13]	*N* = 30 (11 males, 28–73 years)	3.0T, 3D FIESTA, 3D TOF MRA	25/30	The fusion imaging technique of 3D FIESTA and 3D TOF MRA sequences, combining the high anatomical detail provided by the 3D FIESTA sequence with the 3D TOF MRA sequence and its capacity to depict arterial structures, results in a tool that enables quick and efficient visualization and assessment of the relationship between the trigeminal nerve and the neighboring vascular structures.
Han et al [14]	*N* = 40 (15 males, 30–78 years)	1.5T, 3D FIESTA	34/40 (3D FIESTA); 39/40 (3D modeling)	The segmentation and 3D modeling were more accurate than MRI FIESTA in preoperative verification of the NVR and offending vessel.
Ruiz-Juretschke et al [26]	*N* = 74 (22 males, mean age of 61.1 years)	1.5T, DRIVE	64/74	Contingency table analysis for MRI and surgical diagnosis of NVC shows that of the 64 cases diagnosed by MRI, one case was not confirmed by surgery; this patient, therefore, represents a false positive. In contrast, surgery detected NVC in 3 of the 10 patients found not to be affected according to MRI findings; these patients represent false negatives. Therefore, the sensitivity and specificity of 3D T2-weighted MRI were 95% and 87%, respectively.
Yang et al [31]	*N* = 65 (22 males, 35–80 years)	1.5T, 3D CISS, 3D FLASH, 0.1 mmol/kg Gadopentetic acid	54/65 (3D-FLASH); 55/65 (3D-CISS); 60/65 (3D-FLASH + 3D-CISS)	The accuracy and positive rates of the 3D-FLASH + CISS imaging (98.46%, 92.31%) in judging the symptomatic side according to the presence of vascular contacts were higher than those of 3D-CISS (90.77%, 84.62%) or 3D-FLASH (89.23%, 83.08%) sequence.
Xiong et al [29]	*N* = 97 (47 males, 36–74 years)	3.0T or 1.5T 3D CISS	93/97	The 3D relationship between visible structures seen on MRI was consistent with the intraoperative findings in all patients. All cases were divided into three groups by the degree of trigeminal nerve encroachment by SPV. Statistical analysis revealed that the distance from the SPV to the trigeminal nerve was significantly different among the three groups. The diameter of the SPV also differed among the three groups.
Müller et al (2020)	*N* = 48 (32 males, mean age of 59.6 years)	3.0T or 1.5T bSSFP, 3D TOF MRA	8/10 (bSSFP); 31/38 (bSSFP + 3D TOF MRA)	Overall, the MRI findings correctly predicted the intraoperative findings in 91.7% of the 48 patients. The percentage of correct prediction increased from 80 to 94.7% when TOF angiography was adjoined.
Liao et al [18]	*N* = 82 (36 males, 35–72 years)	3.0T, 3D bFFE, 3D MRA, 3D T1w FFE, subtraction images	80/82 (3D bFFE, 3D MRA, 3D T1w FFE); 81/82 (3D bFFE, 3D MRA, 3D T1w FFE + subtraction images)	MR subtraction exhibited greater accuracy than the conventional method in terms of the estimated severity of conflict (87.80% vs. 57.32%), and demonstrated better consistency with surgical findings (k = 0.794 vs. k = 0.365).
Pham et al [25]	*N* = 33 (17 males, mean age of 56.3 years)	3.0T, 3D SPACE, 3D TOF MRA	31/33	The fused D3-SPACE and 3D-TOF-MRA images are highly effective tools for the evaluation and treatment planning of NVC in patients.
Hu et al [15]	*N* = 73 (24 males, 38–77 years)	3.0T or 1.5T 3D FIESTA, 3D TOF MRA	69/74	3D TOF MRA and FIESTA show an overall good ability to predict specific offending vessels. The intra-observer and interobserver variability of NVC measurement for the symptomatic side showed strong agreement with ICC of 0.95 and ICC of 0.90, respectively. For the asymptomatic side, the ICC of the intra-observer and interobserver variability was 0.92 and 0.94.
Wamasing et al [34]	*N* = 60 (16 males, 29–90 years)	3.0T sequences: 3D CISS, 3D SPACE	N/A	SPACE provided better images than CISS for evaluating the trigeminal nerve and prepontine cistern vascularity, indicating a valuable sequence for TN diagnosis

*CISS* constructive interference in the steady state, *DTPA* gadolinium diethylenetriaminepentaacetate, *FIESTA* fast imaging employing steady state acquisition, *FISP* fast imaging with steady precession, *FLASH* fast low angle shot, *MRA* magnetic resonance angiography

* Administered dosage unknown

**Table 2:** Risk of bias classification for diagnostic studies using the QUADRAS-2 tool

	Risk of bias				Applicability concern		
Author (year)	Patient selection	Index test	Reference standard	Flow and timing	Patient selection	Index test	Reference standard
Yamakami et al [30]	High	Low	Low	Unclear	High	Low	Low
Akimoto et al [9]	Low	Low	Unclear	Unclear	Low	Low	Low
Patel et al [24]	Low	Low	Low	Low	Low	Low	Low
Zerris et al [33]	Low	Low	Unclear	Low	Low	Low	Low
Chávez et al (2005)	Low	Low	Unclear	Unclear	Low	Low	Low
Anderson et al [10]	Low	Low	Low	Low	Low	Low	Low
Miller et al [20]	Low	Low	Low	Low	Low	Low	Low
Miller et al [21]	Low	Low	Unclear	Low	Low	Low	Low
Ni et al [23]	Low	Low	Low	Low	Low	Low	Low
Satoh et al [27]	Low	Low	Low	Low	Low	Low	Low
Leal et al [76]	Low	Low	Low	Low	Low	Low	Low
Cha et al [11]	Low	Low	Unclear	Low	Low	Low	Low
Vergani et al [28]	Low	Low	Low	Low	Low	Low	Low
Zeng et al [32]	Low	Low	Low	Low	Low	Low	Low
Lee et al [17]	Low	Low	Low	Low	Low	Low	Low
Maarbjerg et al [19]	Low	Low	Low	Low	Low	Low	Low
Docampo et al [13]	Low	Low	Unclear	Low	Low	Low	Low
Han et al [14]	Low	Low	Low	Low	Low	Low	Low
Ruiz-Juretschke et al [26]	Low	Low	Low	Low	Low	Low	Low
Yang et al [31]	Low	Low	Unclear	Low	Low	Low	Low
Xiong et al [29]	Low	Low	Unclear	Low	Low	Low	Low
Müller et al (2020)	Low	Low	Low	Low	Low	Low	Low
Liao et al [18]	Low	Low	Unclear	Low	Low	Low	Low
Pham et al [25]	Low	Low	Low	Low	Low	Low	Low
Hu et al [15]	Low	Low	Low	Low	Low	Low	Low
Wamasing et al [34]	Low	Low	Low	Low	Low	Low	Low

**Table 3:** Recommendations with regard to MRI examination of patients suspected of classical trigeminal neuralgia and secondary trigeminal neuralgia

Recommendations	
1	T1w and/or T2w imaging of the brain should be carried out to assess structural lesions causing secondary trigeminal neuralgia. This assessment could be optimized by adding a FLAIR sequence of the brain.
2	For a detailed assessment of the cerebellopontine angle, it is recommended to use three-dimensional, thin gradient recalled echo sequences (e.g., 3D CISS, 3D FLASH).
3	A 3D TOF MRA sequence (after presaturation) can be used for the visualization of most vessels, although high-flow vessels (i.e., arteries) are visualized most optimally. Albeit, only limited evidence exists that warrants the addition of a contrast-enhanced T1w sequence to the standard imaging protocol for the visualization of relatively low-flow vessels (i.e., veins).
4	The standard imaging protocol can be carried out at 1.5-T MRI scanning systems. When available and if needed, complementary imaging on a 3.0-T imaging system could be performed.
5	Various studies have shown the added value of DTI in increasing the specificity of MRI in the diagnosis of trigeminal neuralgia. Adding a DTI sequence (e.g., simultaneous multi-slice DTI with readout-segmented echo planar imaging) to the standard MRI protocol could help to optimize radiological imaging of trigeminal neuralgia patients.
6	Although not classified as secondary trigeminal neuralgia according to the ICHD-3 criteria, radiologists can use MRI to help find other causes of trigeminal pain in patients (e.g., nerve inflammation, post-traumatic nerve injury, signs of denervation, dental causes).
7	MRI interpretation should preferably be performed by specialized neuroradiologists.
8	MRI findings need to be clinically integrated and not used as absolute diagnostic criteria.

*CISS* constructive interference in the steady state, *DTI* diffusion tensor imaging, *FLASH* fast low angle shot, *ICHD-3* International Classification of Headache Disorders third edition, *MRA* magnetic resonance angiography, *TOF* time of flight

#### When does a contact turn into a conflict?

Although a neurovascular conflict is frequently observed in TN patients, vascular loops near the trigeminal nerve are also common in asymptomatic individuals or on the contralateral side [19, 39, 40]. Studies therefore aimed to identify features that distinguish a neurovascular conflict from mere neurovascular contact. Proximity to the root entry zone (≤ 3 mm), nerve displacement, and atrophy were consistently associated with classical TN and have been termed “severe neurovascular conflict” [11, 21, 23, 41–60]. These features were not linked to other clinical variables such as age or disease duration [19]. One study even suggested that computational fluid dynamics, assessing vessel wall shear stress, may help identify the pain-inducing vessel [61]. Arteries are the most frequent offending vessels, especially the superior and anterior inferior cerebellar arteries, though other vessels, including the posterior cerebral, basilar, and vertebral arteries (e.g., dolichoectasia), may also be involved [48, 62–64]. Venous neurovascular conflicts, typically involving the superior petrosal vein, have also been reported and may be associated with a shorter trigeminal nerve [11, 16–18, 22, 29, 36, 65–68]. A retrospective study found that if < 35% of the trigeminal nerve is visualized on MRI, neurovascular conflicts may be missed, warranting endoscopic evaluation [69]. Despite the high predictive value of preoperative MRI [62, 70, 71], the absence of a neurovascular conflict on imaging should not preclude neurosurgical assessment [5, 17, 71]. In rare cases, structural lesions other than a neurovascular conflict or demyelination can cause TN, including arachnoid adhesions, bony protrusions, cholesteatomas [72–74], and tumors in the cerebellopontine angle (e.g., epidermoid cysts, meningiomas, schwannomas), observed in about 10% of TN patients [75] Recommendation 2 + 3; Table 3.

#### Microstructural and morphological changes of the trigeminal nerve

Diffusion-weighted and diffusion tensor imaging (DWI/DTI) studies consistently show increased apparent diffusion coefficient values (ADC: normative value approximately 1.525 × 10⁻³ mm²/s ± 0.106 × 10⁻³ mm²/s) and decreased fractional anisotropy values (FA: normative value approximately 0.493 ± 0.069) in the affected trigeminal nerve, indicating microstructural disintegration [76–91]. FA reductions are most pronounced at the neurovascular conflict site and root entry zone in classical TN [92, 93], and similar patterns are seen in secondary TN adjacent to demyelinating plaques [93, 94]. However, Wilcox et al found no such changes in TN patients, but did observe alterations in patients with trigeminal neuropathy [95]. In turn, normalization of DTI metrics after successful treatment has been reported [82–84, 87, 96]. Microstructural alterations are associated with trigeminal nerve atrophy [77, 83, 86] and morphologic changes, such as decreased volume and pontine angle [85, 86, 97–107]. Koh et al demonstrated consistency of DTI metrics at 3 T across protocols, supporting future clinical implementation [108]. A 7 T DTI study, however, found no microstructural changes in the brainstem trigeminal nuclei of TN patients [81] Recommendation 5; Table 3.

#### A crowded fossa might increase the identification of a causative neurovascular conflict

Although the pathophysiology of TN remains partially elusive, neurovascular conflicts induced focal demyelination, resulting in artificial synapse formation and ephaptic signal conduction, are believed to be the cause [109]. However, as described in previous paragraphs, the correlation between neurovascular conflicts and the occurrence of TN is not as straightforward as believed, and various studies shifted focus to other factors that might, at least in part, contribute to the development of TN. One hypothesis in the current literature handles that a reduced posterior fossa volume, especially a reduced volume of the cisterns, might contribute to the (re)occurrence of a neurovascular conflict [110–119], although some conflicting results are presented in the literature [58] (Recommendation 1 + 2; Table 3).

#### Who should be reading the images and where?

Although the attempt to discern neurovascular conflict from neurovascular contact on MRI images has been found useful in a variety of reading studies, one reading study reported that most cases remain ambiguous. Even more, they reported that high-resolution MRI data does not predict the diagnosis of classical TN with an average reading performance of 0.57 per patient. This indicates that reading performance is no better than chance and, therefore, diagnostic imaging should not limit further treatment options [6]. However, in this study, two out of the four readers were neurosurgeons, and one of the readers was a neurosurgery resident, which raises the question of whether experience and specialty have an impact on the diagnostic accuracy. To answer this question, Singhal and Danks undertook another reading study in which they found that radiologists had a strong tendency to underreport a neurovascular conflict on preoperative MRI compared to the neurosurgery readers. However, in this retrospective study, all patients underwent microvascular decompression surgery after the imaging session. The imaging reports from the radiology readers were created prior to the indication of any neurosurgical intervention, whereas neurosurgical reading of the images was carried out in the knowledge of the upcoming neurosurgical intervention. This should be seen as an important methodological shortcoming of the study, which could have introduced information bias [120]. Nevertheless, one other study also found that MRI had a low sensitivity with regard to observing pain-causing neurovascular conflicts. In addition to this, poor interrater reliability and the lack of association between MRI findings and post-operative pain relief resulted in the recommendation that MRI should not prevent the pursuit of microvascular decompression surgery [121]. In another study, the influence of practice settings was discussed in light of the gold standard of microvascular decompression endoscopy. This study showed that the frequency of vascular compression reported by non-academic radiologists was lower than what is reported by academic neuroradiologists reading the same MRI scans [122] (Recommendation 7 + 8; Table 3).

### Secondary TN

If no neurovascular conflict is observed, MRI plays a key role in detecting alternative causes of secondary TN, which include demyelination, ischemia, neoplasms, and other structural lesions [123–126]. Secondary TN is most commonly seen in multiple sclerosis, where signal abnormalities in the cisternal trigeminal nerve or root entry zone are seen in 3–23% of patients [127, 128], though these do not consistently correlate with clinical symptoms.

Several studies have aimed to characterize symptomatic multiple sclerosis lesions. These are often unilateral (79%), involve multiple subunits of the trigeminal nuclear complex, and predominantly affect the spinal and principal sensory nuclei [129, 130]. A distinctive feature is a linear plaque in the intrapontine fascicular part of the nerve. Mousavi et al reported that 66% of MS patients with TN had lesions in the sensory pathways, which were associated with treatment resistance [131]. Other studies corroborated the relevance of intrapontine tract lesions [132, 133], and one study showed that MRI abnormalities may precede TN symptoms by years [7]. Trigeminal contrast enhancement was observed in ~3% of MS patients, suggesting subclinical demyelination [127, 134]. The role of neurovascular conflict in MS-related TN remains debated. One study showed microvascular decompression relieved pain in 67% of MS patients [135], while another found no benefit and discouraged the procedure [136] (Recommendation 1; Table 3).

Although not classified as secondary TN by ICHD-3, MRI can help identify other trigeminal pain causes, including post-traumatic changes, denervation inflammation and dental pathology visible on standard MR sequences [73, 137–145] (Recommendation 6; Table 3).

General recommendations with regard to the radiological imaging of patients suffering from TN (i.e., classical or secondary TN following the ICHD-3 criteria) can be found in Table 3.

## Discussion

This systematic literature review shows the potential pearls and pitfalls of imaging TN patients. Although various groups investigated the potential of MRI for the identification of neurovascular conflicts for the diagnosis of classical TN in patients, this literature review shows that (1) identification of a neurovascular conflict is no proof of the diagnosis classical TN, and (2) the absence of a neurovascular conflict is no proof of the absence of the diagnosis classical TN. Phenotypical characteristics of the neurovascular conflict (i.e., nerve atrophy, nerve deviation and a neurovascular conflict at the trigeminal root entry zone) increase the specificity of the use of MRI for the diagnosis of classical TN. Furthermore, a decreased volume of the posterior fossa might contribute to elucidating a causative neurovascular conflict. Contrary to neurovascular conflicts, the use of MRI for the diagnosis of secondary TN seems to be more reliable, as some distinctive features of white matter lesions (i.e., lesions at the level of the one or more subunits of the trigeminal nuclear complex or a linear lesion involving the intrapontine fascicular part of the trigeminal nerve) have been described as indicative of secondary TN. However, much debate exists with regard to the imaging protocol that needs to be used in these patients. Based on the available literature, some recommendations are provided for the reader at the end of the manuscript.

As highlighted by the current review, it is now well recognized that in TN, MRI enables the detection and characterization of potential causative factors. Next to the aforementioned non-specific neurovascular conflicts, a crowded posterior fossa and specific white matter lesions, other hypotheses exist describing from which anatomical findings TN arises. For example, one hypothesis states that a flattened Meckel’s cave may cause TN [146, 147], which is in line with the results showing a correlation between Meckel’s cave shape and percutaneous balloon compression outcomes [148, 149]. Other authors hypothesized that a narrow oval foramen might contribute to the development and recurrence of TN involving the third branch of the trigeminal nerve [150]. Although these theories are interesting, there is no pathophysiological theory that consistently links these anatomical variants to the development of TN. Histopathological studies might bridge this gap between findings on MRI and the development of TN. One of the key findings in the histopathological evaluation of TN is the presence of demyelination in the trigeminal nerve root. Zimering et al reported on a case showing that ectopic brain tissue in the trigeminal nerve could lead to focal demyelination, which may result in ephaptic conduction, a phenomenon similar to that seen in primary TN en secondary TN [151]. Another study emphasized that arterial compression of the trigeminal nerve is a prevalent cause of TN, and histological examination can reveal the extent of nerve damage due to such compression [152]. Moreover, studies have shown that histopathological findings can correlate with imaging results. For example, Neetu et al noted that DTI findings align with histopathological findings of demyelination and axonal changes [94]. Therefore, such integration of imaging and histopathological data enhances the understanding of the pathophysiological mechanisms driving TN and might lead to more specific diagnostic methods and novel, targeted therapeutic approaches.

This systematic review has its strengths and limitations. Strengths include the rigorous methodology and the large number of retrieved and included articles. Another strength is the low risk of bias and the low applicability concern of the retrieved literature. This indicates that the literature discussed in this systematic review is of high quality and the reviewed papers had research questions and study populations that are applicable on answering the research question of the present systematic review. One of the key limitations, however, is that the research on imaging primary and secondary trigeminal neuralgia is heterogeneous, using different study designs. Consequently, included in this review are technical validation studies, protocol optimization studies, diagnostic accuracy studies, observer performance studies and retrospective cohort imaging studies. Next to these different designs, studies used a variety of MRI sequences at different field strengths, and disease states are classified differently as positive or negative. This heterogeneity precluded a meaningful meta-analysis of (subsets of) the included articles. However, despite the heterogeneity of the available studies, we were able to extract several key recommendations for the radiological workup of TN. These recommendations (summarized in Table 3) emphasize the importance of high-resolution 3D T2-weighted sequences, the potential added value of DTI, and the need for interpretation by experienced neuroradiologists. They directly reflect the main limitations and opportunities identified in this review.

## Conclusion

This systematic review highlights the role of MRI in the diagnostic workup of TN, particularly in detecting clinically relevant neurovascular conflicts. Furthermore, we distilled recommendations that provide a practical framework for radiologists and other clinicians, but should be regarded as guidance that must be integrated with the clinical context rather than as absolute diagnostic criteria. More specifically, high-resolution 3D sequences and 3.0-T MRI improve visualization of neurovascular anatomy, while DTI offers insight into microstructural nerve changes. These modalities show potential in distinguishing classical from secondary TN. However, challenges remain due to overlap with findings in asymptomatic individuals, moderate diagnostic accuracy, and variable interobserver agreement. Despite these limitations, MRI remains a valuable tool—especially when integrated with clinical assessment. Future studies should validate standardized protocols in larger cohorts to refine diagnostic accuracy and support clinical decision-making.

## Abbreviations

CISS: Constructive interference in the steady state
DTPA: Gadolinium diethylenetriaminepentaacetate
FIESTA: Fast imaging employing steady state acquisition
FISP: Fast imaging with steady precession
FLASH: Fast low angle shot
MRA: Magnetic resonance angiography
MRI: Magnetic resonance imaging
TN: Trigeminal neuralgia
